# Catalytic performance and deactivation of Ni/MCM-41 catalyst in the hydrogenation of pure acetylene to ethylene

**DOI:** 10.1039/c9ra09878j

**Published:** 2020-01-09

**Authors:** Shuzhen Zhou, Lihua Kang, Zhu Xu, Mingyuan Zhu

**Affiliations:** School of Chemistry and Chemical Engineering of Shihezi University Shihezi Xinjiang 832003 P. R. China zhuminyuan@shzu.edu.cn +86 9932057210 +86 9932057270; Key Laboratory for Green Processing of Chemical Engineering of Xinjiang Bingtuan Shihezi Xinjiang 832003 P. R. China

## Abstract

Ni/MCM-41 catalysts were prepared by an impregnation method for acetylene hydrogenation to ethylene based on the calcium carbide acetylene route. X-ray diffraction and transmission electron microscopy indicated that Ni was uniformly dispersed on the support. Temperature-programmed reduction and X-ray photoelectron spectroscopy demonstrated a strong interaction between Ni and MCM-41, and Ni(0) and Ni(ii) coexisted in the catalyst. We optimized the catalytic activity by optimizing the Ni loading and reaction conditions including temperature, space velocity, and hydrogen/acetylene ratio. The acetylene conversion reached 100%, the ethylene selectivity reached 47%. Additionally, we tested the catalyst stability; the acetylene conversion was maintained at 100% for 25.73 h and was then rapidly reduced. ICP, TEM, FT-IR, thermogravimetric analysis and BET were used to investigate the reasons for catalyst deactivation; it was found that green oil deposition on the catalyst surface was the main reason for the catalyst deactivation.

## Abstract 2

Ni/MCM-41 catalysts were prepared by an impregnation method for acetylene hydrogenation to ethylene based on the calcium carbide acetylene route.

## Graphical abstract

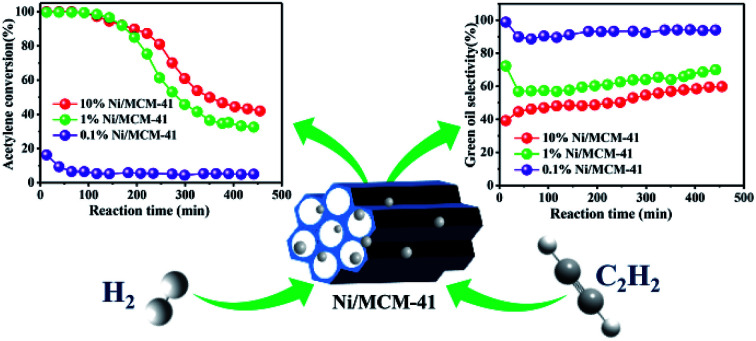

## 1. Introduction

**Scheme 1:**
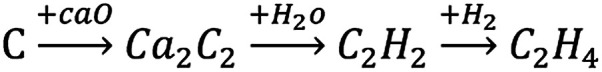
Calcium carbide acetylene route to ethylene.

Ethylene is an important petrochemical raw material, which can be used to synthesize fibers, rubber, plastics, and other materials. Its production is mainly based on cracking of hydrocarbons *via* the petrochemical route to produce ethylene. However, as oil reserves shrink, it has become economically more feasible to produce ethylene based on the calcium carbide acetylene route (Scheme 1) in areas with abundant coal resources, especially in China, which is rich in coal and poor in oil. However, for acetylene hydrogenation, the main research involved removing 0.1–1% of acetylene from the ethylene-rich stream, to avoid deactivation of the catalyst in the downstream production process.^1,2^ To the best of our knowledge, studies on pure acetylene (C_2_H_2_ > 99.99% with minimal H_2_S and PH_3_) hydrogenation to ethylene are rare. Therefore, the hydrogenation of calcium carbide acetylene to ethylene has important research significance.

Although the reaction system is different, the trace acetylene hydrogenation catalyst system can provide a reference for pure acetylene hydrogenation. Since Pd exhibits excellent hydrogenation activity, current research mainly focuses on a Pd catalyst, and the catalytic activity is improved by support modulation^3^ and addition of Ag,^4^ Cu,^5^ and others. Pei *et al.*^5^ prepared Cu alloyed with Pd as a single-atom catalyst for semi-hydrogenation of acetylene with high concentrations of hydrogen and ethylene; the acetylene conversion reached 100% and the ethylene selectivity was 85%. Zhou *et al.*^6^ prepared a PdZn intermetallic nanostructure; due to a good distribution of Pd in Pd–Zn–Pd, the chemical adsorption of ethylene was reduced and excessive hydrogenation of ethylene was inhibited, ethylene selectivity remained at 90% at relatively low temperatures (60 °C), and ethane output was low even at high temperatures (200 °C) when acetylene was completely converted. However, for pure acetylene hydrogenation, due to the high acetylene concentration, a large number of catalytically activity sites are required, so a large amount of catalyst or a high loading are needed, resulting in a high cost. Therefore, Pd-based catalysts may be unsuitable for pure acetylene hydrogenation from an economic perspective.

In recent years, non-precious metal Ni has attracted much attention in acetylene hydrogenation.^7,8^ Chen *et al.*^9^ prepared Ni–In bimetallic catalysts to study the effect of In addition on the reaction. Ni/SiO_2_ conversion could be maintained at 100% before deactivation after 2 h. Adding In improved the ethylene selectivity. However, the green oil selectivity exceeded 30%. Green oil contains mainly C4 and C6 hydrocarbons and polymers, and Ahn *et al.*^10^ demonstrated that the precursor of green oil is 1, 3-butadiene. Wang *et al.*^11^ found that by adding Ga, Ni–Ga intermetallic compounds could be formed to improve stability and ethylene selectivity; however, even at the optimal Ni/Ga molar ratio, the green oil selectivity exceeded 15%. Both studies consider green oil formation, and it was suggested that the green oil selectivity exceeds that of ethane. This indicates that the Ni-based catalyst used for acetylene hydrogenation will generate large amounts of green oil as a by-product. To control Ni dispersion, Dai *et al.*^12^ used the metal organic framework ZIF-8 to prepare a single-atom Ni/N–C catalyst for acetylene removal from an ethylene-rich stream, with acetylene conversion and ethylene selectivity both exceeding 90%. However, due to the lower acetylene concentration, they neglected green oil in the reaction calculation. The above studies were all used for acetylene removal from the ethylene-rich stream, and the acetylene concentration was below 2%. In practice, during acetylene hydrogenation, the acetylene concentration strongly affects the green oil selectivity. Trimm *et al.*^13^ prepared a Ni/SiO_2_ catalyst and studied the distribution of acetylene oligomer reaction products with a feed of 25% C_2_H_2_/75% H_2_; they found that approximately half of the acetylene was converted to a liquid oligomer. Therefore, green oil must be considered in the pure acetylene hydrogenation reaction, and as far as we know, almost no one has studied the use of a Ni single metal catalyst in the hydrogenation of pure acetylene to ethylene and discussed the effect of reaction conditions on acetylene conversion. In addition, according to the literature,^14^ amino-functionalized MCM-41 can strengthen the interaction between Ni nanoparticles and MCM-41, reduce particle size, and improve dispersion of nickel particles.

Therefore, in this work, we used amino functionalized MCM-41 to immobilize Ni particles on the support prepared a uniformly dispersed Ni/MCM-41 catalyst and mainly studied the effects of Ni loading, and the reaction conditions (temperature, space velocity, hydrogen/acetylene ratio) for hydrogenation of pure acetylene to ethylene. The stability and regenerative capacity of the 1%Ni/MCM-41 catalyst and the main reason for the catalyst deactivation in this reaction were investigated and discussed. This work provides a strategy for further study of Ni-based catalysts for hydrogenation of pure acetylene to ethylene.

## 2. Experimental

### 2.1 Catalyst preparation

MCM-41 (surface area: 1124 m^2^ g^−1^, average pore diameter: 3.68 nm) was purchased from Nanjing Xianfeng Nano Material Technology Co., Ltd. amino functionalized MCM-41 was obtained by the following steps, was reported in our previous work.^14^ A toluene solution containing 2 g of MCM-41 was uniformly dispersed by ultrasonication for 5 minutes, and after passing through nitrogen for five minutes, 4.5 mL 3-aminopropyltriethoxysilane (APTES) was dropwise added with stirring under nitrogen atmosphere at room temperature. After reacting for 12 h, filtering and washed with deionized water to afford amino functionalized MCM-41.

The MCM-41-supported metallic Ni catalysts were obtained using incipient impregnation method. Amino functionalized MCM-41 (2 g) was added to 30 mL deionized aqueous solution dissolved with a certain amount of nickel nitrate under agitation, and dispersed by ultrasonication for 10 min, and continuously stirred for 24 h, then dried by rotary evaporated at 70 °C after that heat treatment for 4 h at 400 °C in a muffle furnace. The obtained sample was then reduced in a tube furnace under 5% H_2_/Ar (100 mL min^−1^) atmosphere at 500 °C for 4 h to obtain the Ni/MCM-41 catalyst. Catalysts with different Ni contents were obtained by adding different amounts of Ni(NO_3_)_2_·6H_2_O as described above and named *X*%Ni/MCM-41 (*X* represents the Ni load).

### 2.2 Catalyst characterization

X-ray diffraction (XRD) patterns were obtained using Cu-Kα irradiation (*λ* = 0.15406 nm) at 40 kV on a Bruker D8 Advance X-ray diffractometer. Temperature-programmed reduction (TPR) signal was collected on a Quantachrome Instruments automated chemisorption analyzer. First, about 20 mg of calcined samples were added into a U-shaped quartz tube, after remove the adsorbed water, the catalyst was heated to 900 °C at 10% H_2_/Ar atmosphere. Transmission electron microscopy (TEM) images were recorded by Tecnai F30 field emission transmission electron microscope (300 kV) at room temperature. X-ray photoelectron spectrum (XPS) was tested on a Thermo Fisher Scientific ESCALAB 250Xi X-ray photoelectron spectroscopy analyzer with a monochrome Al-Kα X-ray excitation source; the binding energy of C1s was set as 284.8 eV for calibration. A Thermo-ICAP 6300 plasma emission spectrometer (USA) was used to obtain the actual Ni content of the catalyst. Fourier transform infrared spectroscopy (FT-IR) results were used to analyze changes in catalyst surface functional groups before and after the reaction, which was performed on a Nicolet Avatar 360 spectrometer. Thermo-gravimetric analysis (TGA) was performed on a Netzsch synchronous thermal analyzer to analyze the carbon deposit on the catalyst. The catalyst was analyzed at 10 °C min^−1^ from 30 °C to 900 °C in an air atmosphere.

### 2.3 Catalyst activity test

We produced ethylene through the calcium carbide acetylene route and only acetylene (>99.99%) and hydrogen (>99.99%) were fed into the reaction tube without ethylene or other equilibrium gases. All gas flow in the reaction was controlled with a mass flow controller, and the gas inlet pressure was 0.1 MPa. The reaction was performed continuously on a fixed bed reactor with a diameter of 10 mm stainless steel tube. Before the reaction, the 0.1 g catalyst added into reaction tube and pretreated in 80 mL min^−1^ H_2_ atmosphere at 150 °C for 2 h to remove moisture from the catalyst, and to provide an initial temperature for the reaction. Adjust the hydrogen flow rate to the required value for the reaction, a certain amount of acetylene was introduced to perform the reaction, and the temperature was controlled at a specified reaction temperature (100–300 °C). The reactor space velocity was calculated in the light of the acetylene flow rate. The products were detected online using a Shimadzu GC-2014C gas chromatograph with TCD detector and a Porapak-N column (2.1 mm × 2 m).

The acetylene conversion (*X*) and products selectivity (*S*) were obtained by the following formulas:
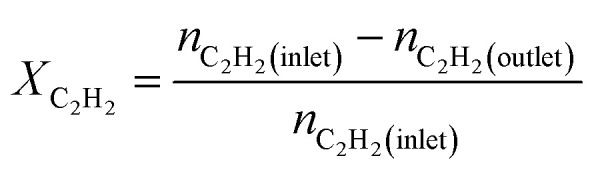

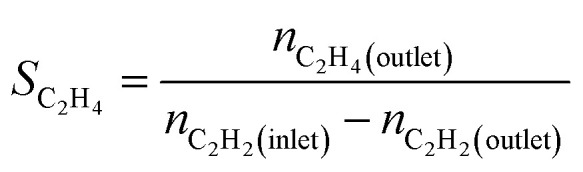

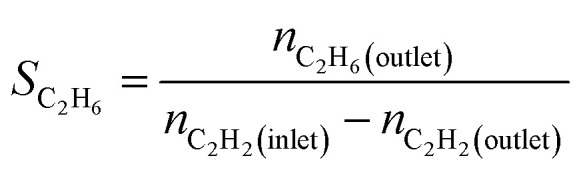
*S*_green oil_ = 1 − *S*_C_2_H_4__ − *S*_C_2_H_6__

In the above formula, green oil represents products other than C_2_.

## 3. Results and discussion

### 3.1 Catalyst characterization results

**Fig. 1:**
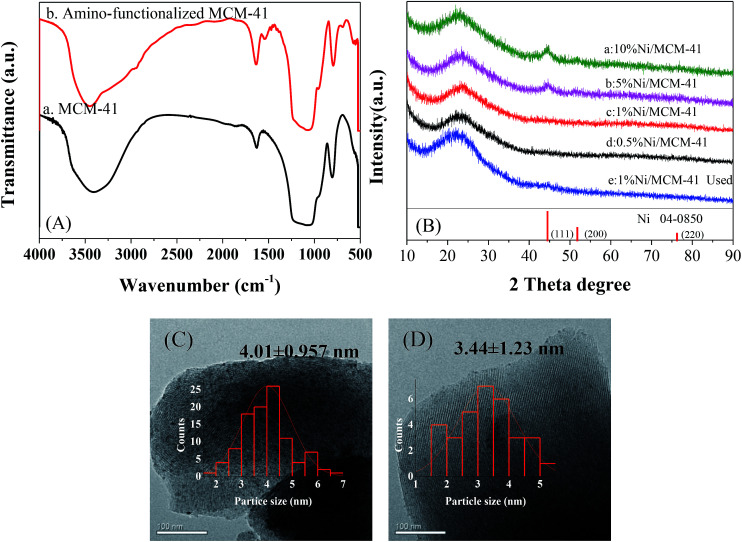
(A) Infrared spectra of MCM-41 before and after modification, (B) XRD patterns of different catalysts, (C) and (D) correspond to TEM images of the 10%Ni/MCM-41 and 1%Ni/MCM-41, respectively.

To ensure the successful preparation of amino functionalized MCM-41, we performed FT-IR on the MCM-41 before and after modification, as shown in Fig. 1(A). Because of the –OH vibration, all the samples showed a characteristic absorption peak at 1630 cm^−1^.^15^ The peaks around 1233 to 1073 and 800 cm^−1^ were ascribed to vibration of the Si–O–Si bond.^16,17^ Significant peaks were observed at 3300 and 1540 cm^−1^ in the infrared spectrum (b) due to the vibration of –NH^+^, indicating the presence of an aminated functional group.^18,19^ Hence, we successfully prepared amino-functionalized MCM-41. We determined the actual Ni loading of the prepared catalyst by ICP in Table 1. The actual Ni loadings were close to the theoretical values, indicating that Ni was successfully loaded into the catalyst. The XRD patterns of *X*%Ni/MCM-41 catalysts after reduction were depicted in Fig. 1(B). The 10%Ni/MCM-41 catalyst displayed three distinct diffraction peaks at 2*θ* = 44.5°, 54.8°, and 76.4° correspond to the (111), (200), and (220) planes of face-centered-cubic Ni, and we calculated the average particle size as about 4.59 nm using the Scherrer equation. The signal of the Ni diffraction peaks decreased with Ni content decrease, and almost no Ni diffraction peaks were observed for 1%Ni/MCM-41 and 0.5%Ni/MCM-41, which may be attributed to Ni existing in an amorphous state or the Ni content being too low to detect when the loading was decreased.^20^ To further investigate the catalyst morphology, we performed TEM, and the results are shown in Fig. 1(C) and (D). In both catalysts, Ni nanoparticles uniformly dispersed on MCM-41. We calculated the average particle sizes of 10%Ni/MCM-41 (Fig. 1(C)) and 1%Ni/MCM-41 (Fig. 1(D)) as 4.01 and 3.44 nm, respectively, which indicated that the Ni nanoparticle size decreased with the loading, which may be due to enhanced interaction between Ni and MCM-41.

**Table 1:** Actual Ni loadings of *X*%Ni/MCM-41

Catalyst	Actual loading	Catalyst	Actual loading
10%Ni/MCM-41	9.7%	0.5%Ni/MCM-41	0.57%
5%Ni/MCM-41	4.4%	0.3%Ni/MCM-41	0.33%
1%Ni/MCM-41	1.1%	0.1%Ni/MCM-41	0.11%

**Fig. 2:**
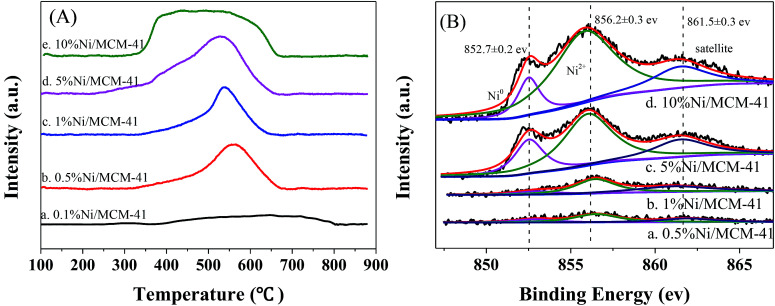
(A) H_2_-TPR profiles for catalysts with different Ni loads, (B) Ni 2p_3/2_ XPS spectra for catalysts with different Ni loads.

To measure reduction ability of the catalyst and the strength of the interaction of the Ni species with MCM-41 surface, we conducted a TPR test on the catalyst after calcination at 400 °C, the results are shown in Fig. 2(A). The 10%Ni/MCM-41 shows a broad peak from 350–650 °C, the peak at about 400 °C ascribed to reduction of large Ni particles, which have no or little interaction with the support, and the peak above 500 °C was put down to reduction of small Ni oxide particles that interacted strongly with support.^21,22^ As the Ni loading decreasing, the reduction peak around 400 °C gradually disappeared, and that around 500 °C gradually moved to higher temperature, consistent with the literature results,^23^ which indicates that the interaction between MCM-41 and Ni becomes stronger and the metal particles become smaller as the load decreases. Meanwhile, as the load decreases, it is more difficult for NiO particles to be reduced to Ni, consistent with the results of TEM and XRD.

To determine the valence state of Ni in the catalysts, we performed XPS characteristic, as displayed in Fig. 2(B). It is clear that the Ni 2p spectra of these four catalysts all can be divided into three peaks, with binding energies of 852.7 and 856.2 eV corresponding to zero-valent metal Ni and Ni oxide with Ni 2p_3/2_, respectively.^24,25^ A satellite peak at 861.5 eV was also observed.^26^ These results indicate that both Ni(0) and Ni(ii) are present in the catalyst. This is consistent with the TPR results; NiO nanoparticles are not completely reduced to Ni nanoparticles at the experimental reduction temperature.

### 3.2 Optimization of Ni content and reaction conditions

**Fig. 3:**
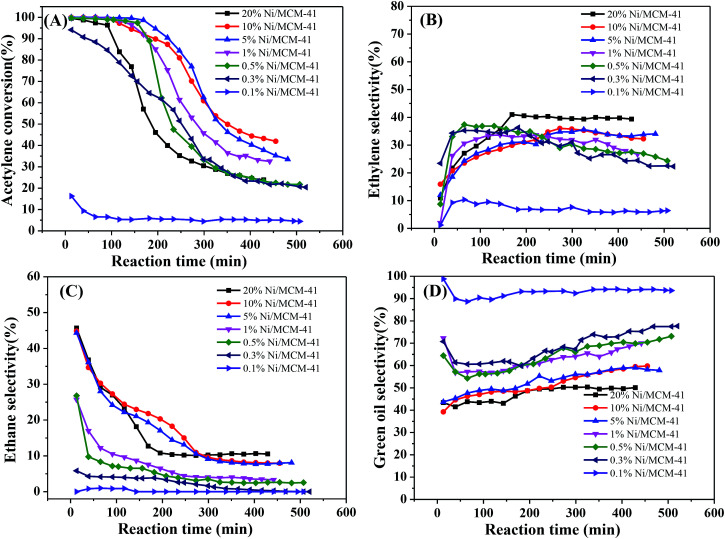
Effect of different Ni content on catalyst performance (reaction conditions: 12 000 mL g^−1^ h^−1^, 250 °C and v(H_2_)/v(C_2_H_2_) = 2); (A) acetylene conversion, (B) ethylene selectivity, (C) ethane selectivity, (D) green oil selectivity.

We initially measured the catalytic performance with different Ni contents. The results are in Fig. 3. As the Ni content increased, the acetylene hydrogenation activity initially increased significantly. When the Ni content reached 0.5%, the initial acetylene conversion had already reached 100%; however, all catalytic performances decreased significantly after a short period of time (Fig. 3(A)), while the ethylene selectivity remained relatively stable, at only about 35% (Fig. 3(B)). The selectivity of ethane decreased with the decrease of Ni content and also decreased as the reaction progressed; for 1%Ni/MCM-41, the ethane selectivity reached 25% at the start of the reaction but dropped to 5% after the reaction was stabilized (Fig. 3(C)). It can also be seen from Fig. 3(D), the lower Ni load, the higher selectivity of green oil, combined with the TPR result indicating that this metal species with strong interaction with the support has a certain contribution to the selectivity of green oil. What's worse, selectivity to the most undesirable by-product green oil exceeded 40%. According to literature reports,^27–29^ the adsorption heat of acetylene on a Ni surface is 150–280 kJ mol^−1^, that for hydrogen is 80 kJ mol^−1^, and the adsorption heat of ethylene is about 100 kJ mol^−1^, which indicates that acetylene is more easily adsorbed on a Ni surface than the others; however, more acetylene and less hydrogen was adsorbed, so that the adsorbed acetylene cannot be completely consumed by hydrogenation, but is polymerized to form oligomers or green oil and deposited on the catalyst surface. As the reaction time increases, the green oil accumulated on the catalyst, might block the catalyst channels and cover the active sites, resulting in catalyst deactivation.^30^

**Fig. 4:**
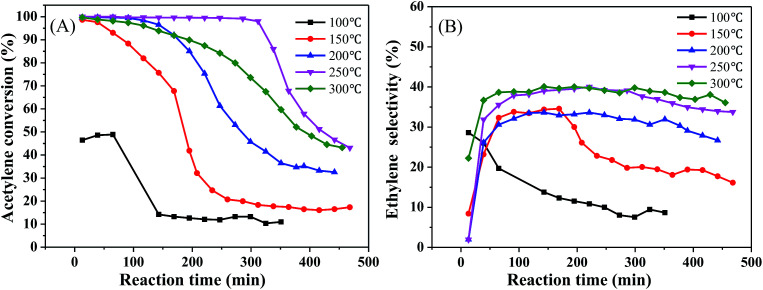
Effect of reaction temperature on catalyst performance (reaction conditions: 12 000 mL g^−1^ h^−1^ and v(H_2_)/v(C_2_H_2_) = 2); (A) acetylene conversion, (B) ethylene selectivity.

To determine the effect of the reaction conditions on the catalytic performance, we selected 1%Ni/MCM-41 for the following experiments. Fig. 4 displays the effect of different reaction temperatures on catalytic activity at 12 000 mL g^−1^ h^−1^ and v(H_2_)/v(C_2_H_2_) = 2. The acetylene conversion increases with temperature, the acetylene conversion can reaches 100% at 200 °C. As the temperature was increased further, the conversion rate of acetylene remained constant. Simultaneously, as the temperature increased, the catalyst improved and was better at 250 °C (Fig. 4(A)). The ethylene selectivity increased with temperature and then remained constant at approximately 40% (Fig. 4(B)), so we chose 250 °C as the temperature for space velocity optimization.

**Fig. 5:**
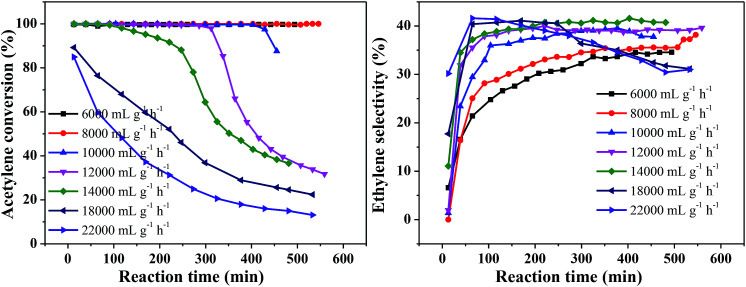
Effect of acetylene space velocity on catalyst performance (reaction conditions: 250 °C and v(H_2_)/v(C_2_H_2_) = 2); (A) acetylene conversion, (B) ethylene selectivity.

Fig. 5 shows the effect of different space velocities on catalytic activity at 250 °C and v(H_2_)/v(C_2_H_2_) = 2. As the space velocity increased from 6000 to 18 000 mL g^−1^ h^−1^, the initial acetylene conversion could reach 100%. However, the catalyst stability was significantly reduced. This may be due to the larger the amount of acetylene treated, the more green oil accumulated on the catalyst surface, resulting in faster deactivation of the catalyst in the same time. Continue increase the space velocity to 22 000 mL g^−1^ h^−1^, the initial acetylene conversion only 85% and rapidly decrease from 85% to 13% within 8 h (Fig. 5(A)). This is because the catalyst does not provide enough active sites for complete conversion of acetylene at high velocity. The ethylene selectivity increased obviously with the increase of space velocity first and then remained at about 41% (Fig. 5(B)), which is consistent with the literature.^31,32^ Considering the stability of the catalyst and the ethylene selectivity, we optimized the hydrogen/acetylene ratio with a space velocity of 8000 mL g^−1^ h^−1^.

**Fig. 6:**
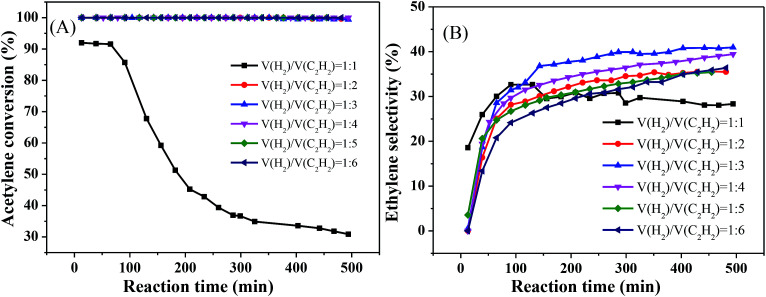
Effect of v(H_2_)/v(C_2_H_2_) on catalyst performance (reaction conditions: 250 °C and 8000 mL g^−1^ h^−1^); (A) acetylene conversion, (B) ethylene selectivity.

Fig. 6 reveals the effect of different volume ratios of hydrogen to acetylene on the activity of the catalyst at 250 °C and 8000 mL g^−1^ h^−1^. As the ratio of hydrogen to acetylene increased, the conversion rate of acetylene tended to increase. When the ratio of hydrogen to acetylene reached 2, the conversion rate of acetylene reached 100%. When the ratio of hydrogen to acetylene was increased further, there was no change in the acetylene conversion, and no significant inactivation occurred after reacting for 8 h (Fig. 6(A)). However, unlike the acetylene conversion, the ethylene selectivity increased gradually with the volume ratio of hydrogen to acetylene and then decreased (Fig. 6(B)), which is consistent with the literature.^33,34^ This is because, as the amount of hydrogen increases, a large amount of hydrogen competitively adsorbs on the catalyst surface with acetylene, which reduces the acetylene adsorption on the catalyst surface, thus inhibiting green oil formation and improving the stability. However, when the amount of hydrogen reaches a certain value, the undesorbed ethylene which is more easily further hydrogenated to ethane, reducing ethylene selectivity. The ethylene selectivity reached 40.96% at the optimum volume ratio of hydrogen to acetylene.

Finally, we obtained the optimal reaction conditions: *T* = 250 °C, 8000 mL g^−1^ h^−1^, and v(H_2_)/v(C_2_H_2_) = 3.

### 3.3 Catalyst stability test and regenerative capacity

In the industrial process, it is crucial to consider the regenerative capacity of the catalyst from an economic perspective. Hence, we tested the stability of the catalyst and investigated its regenerative capacity. The regeneration process was as follows: after each test catalyst deactivation, the catalyst was regenerated by passing H_2_ (80 mL min^−1^) through the reaction tube at 500 °C for 4 h and then continuing to pass acetylene to react under the same conditions.

**Fig. 7:**
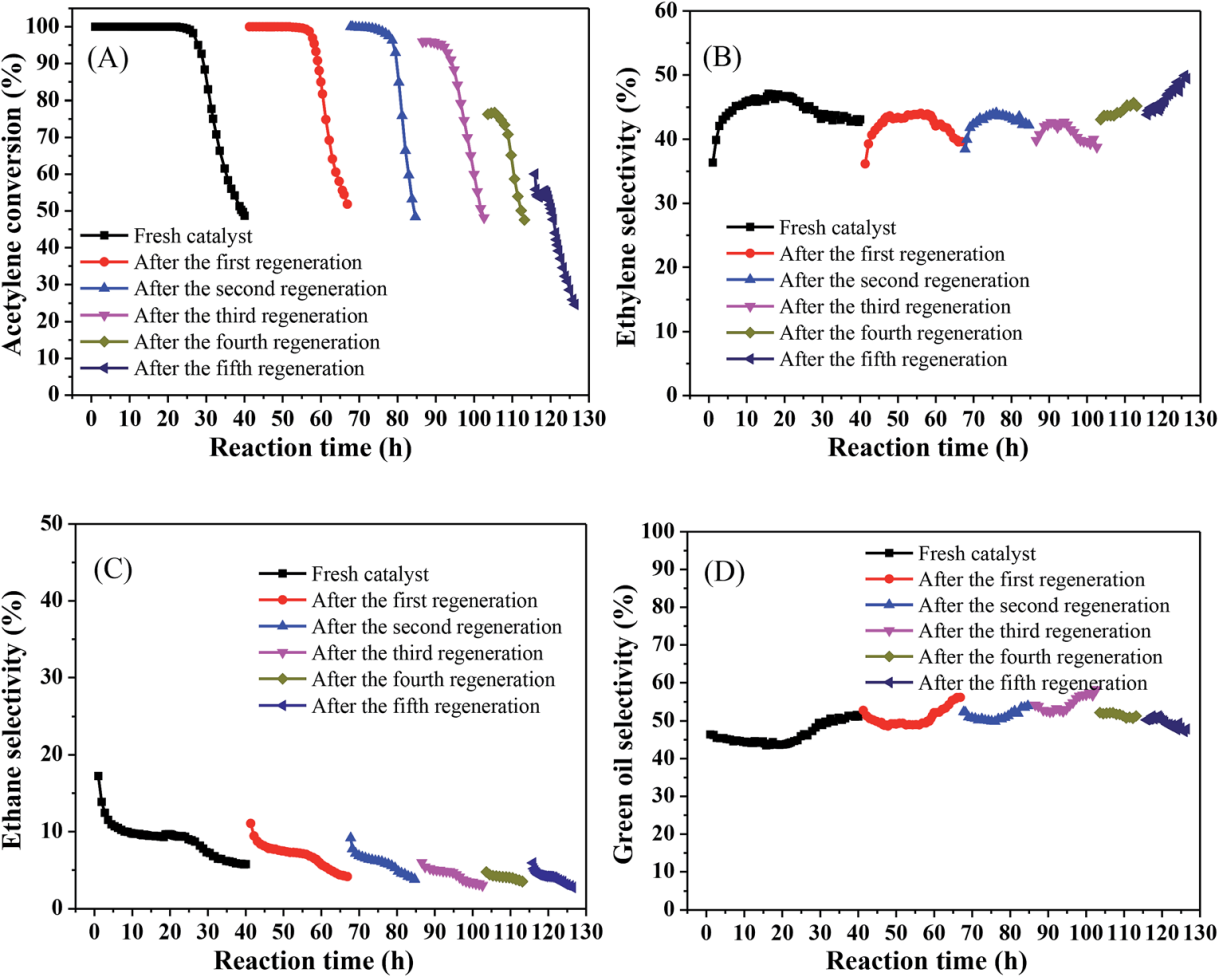
Catalyst stability test and regeneration of the 1%Ni/MCM-41 (reaction conditions: 250 °C, 8000 mL g^−1^ h^−1^, and v(H_2_)/v(C_2_H_2_) = 3); (A) acetylene conversion, (B) ethylene selectivity, (C) ethane selectivity, (D) green oil selectivity.

Fig. 7 shows that the acetylene conversion over the fresh catalyst was maintained at 100% for 25.73 h and was then rapidly reduced from nearly 100% to 48% in the subsequent 14.3 h (Fig. 7(A)), which may be due to green oil accumulating on the catalyst surface during the reaction, gradually blocking the catalyst pores and covering the Ni active sites, resulting in rapid deactivation of the catalyst. As the reaction progressed, the ethylene selectivity increased from 36% to 47% and then slowly decreased to about 43%. In all subsequent regeneration experiments, the product selectivity remained relatively stable, with an ethylene selectivity of about 45% (Fig. 7(B)), ethane selectivity below 10% (Fig. 7(C)), and green oil selectivity exceeding 40% (Fig. 7(D)). However, the catalyst stability showed a different trend. The initial activity of the catalyst reached 100% after the first regeneration and remained stable for a considerable period of time (15.77 h), which may be because, after H_2_ treatment, part of the green oil decomposed so that the pores were reopened and the active sites were exposed, resulting in recovery of acetylene conversion, which is consistent with the results observed by previous research.^35,36^ As the regeneration times increased, the stabilization time of the catalyst decreased slowly and the catalytic activity decreased. After the second regeneration, this stabilized above 99% for 7.97 h. However, after the third regeneration, the initial activity of the catalyst was only 96%, and the catalyst was rapidly deactivated after stabilization above 91% for 8.4 h. After each of subsequent regeneration, the activity and stability of the catalyst gradually worsened and even the initial activity did not reach 70% after the fifth regeneration. This may be due to hydrocarbons that cannot be removed by regeneration; as the number of regenerations increases, they may constantly accumulate, progressively shortening the life of the catalyst after regeneration so that it eventually fails to achieve its initial activity and rapidly deactivated.

### 3.4 Investigation of catalyst deactivation

**Fig. 8:**
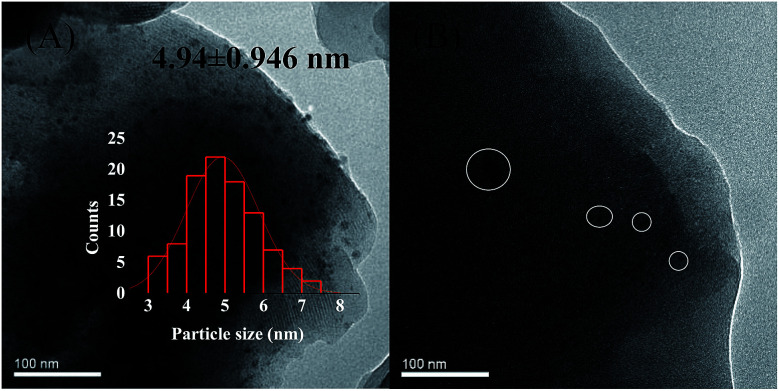
TEM images of the (A) 10%Ni/MCM-41 after reaction 7.5 h, (B) 1%Ni/MCM-41 after reaction 126 h.

Deactivation of the catalyst is mainly attributing to poisoning, carbon deposition, aging sintering, and loss of active components. However, it was reported that green oil and carbon deposition are the main reason for catalyst deactivation during the acetylene hydrogenation.^37^ To investigate the cause of catalyst deactivation, we characterized the catalyst after the reaction. We initially performed an ICP test on the catalyst after the reaction. To eliminate the influence of carbon deposition as far as possible, the catalyst was roasted at 650 °C for 3 h in air before the test, and the Ni loading of the 1%Ni/MCM-41 catalyst after the reaction was measured as 0.90%, indicating that catalyst deactivation was not caused by loss of active components. The XRD pattern of the catalysts after the reaction 126 h is shown in Fig. 1(B). It was found that the diffraction peak of Ni was still not visible in the catalyst 126 h after reaction, just as in the fresh catalyst, which may be due to the low Ni load or the absence of severe agglomeration of the particles after reaction. Fig. 8 displayed the TEM images of the catalysts after the reaction. The average Ni nanoparticle size for 10%Ni/MCM-41 (Fig. 8(A)) catalyst was 4.94 nm, which was slightly higher than the fresh catalyst (Fig. 1(C)). For 1%Ni/MCM-41, after the 126 h regeneration reaction, only some of the particles were significantly agglomerated, but other Ni nanoparticles were still present in the form of small particles (Fig. 8(B)), which indicated that the agglomeration of nickel nanoparticles was not the reason for catalyst deactivation rapidly.

**Fig. 9:**
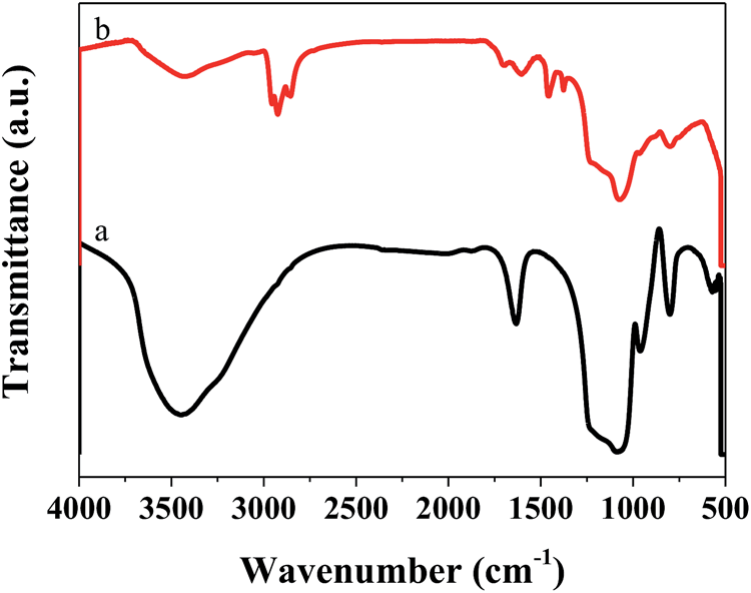
FT-IR spectra of 1%Ni/MCM-41 before (a) after reaction 126 h (b).

We also performed FT-IR on the catalyst after the reaction, and the results are displayed in Fig. 9. Compared with the catalyst before the reaction, that after the reaction showed many different characteristic peaks. The absorption peaks at 2854 and 2924 cm^−1^, and 2869 and 2955 cm^−1^, were ascribed to vibration of the –CH_2_ and –CH_3_ groups, respectively.^38,39^ The peak at 1456 cm^−1^ was ascribed to the C=C group, indicating that C=C was present in the catalyst after the reaction.^40^ These results indicated the presence of aliphatic coke on the catalyst surface. As this was soft coke, it could be removed after regeneration, which is why the catalyst could regain some of its activity.

**Fig. 10:**
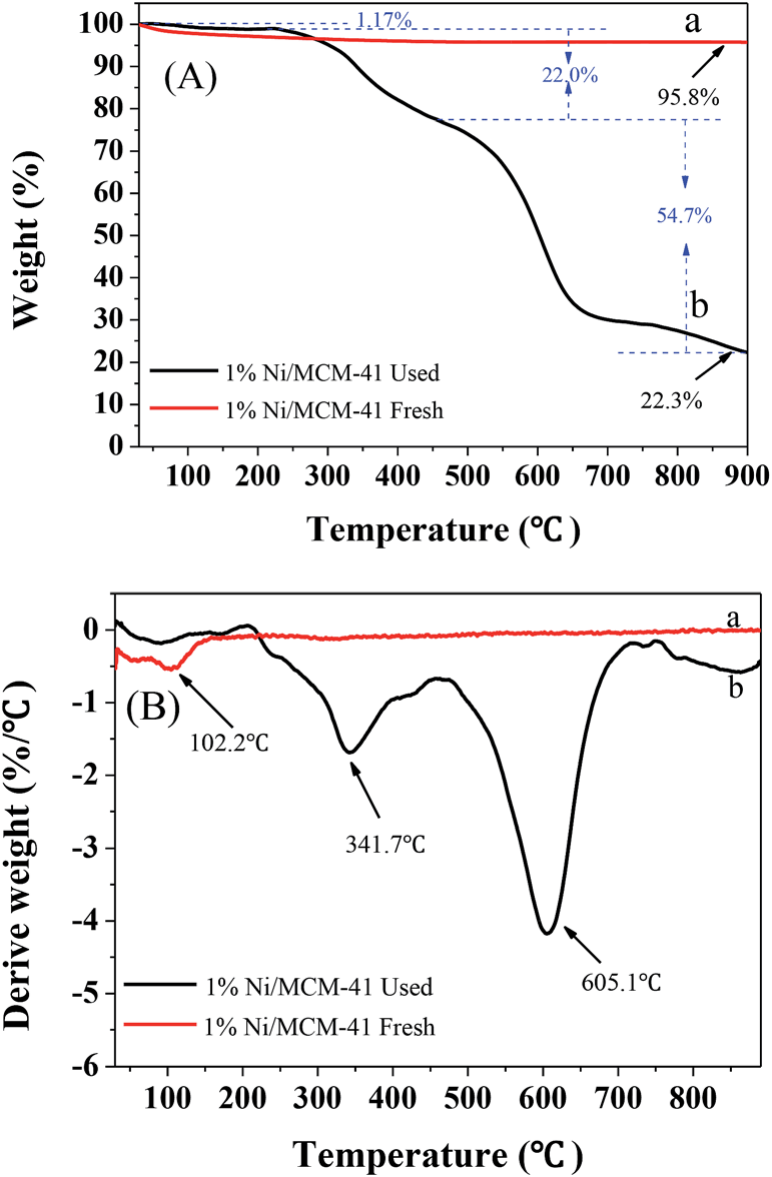
TG (A) and DTG (B) curve of 1%Ni/MCM-41 fresh (a) and after reaction 126 h (b).

To further determine the amount of hydrocarbon deposition on the catalyst surface after the reaction, TG-DTG characterization of the 1%Ni/MCM-41 catalyst before and after the reaction was performed, as shown in Fig. 10. Both samples showed significant weight loss around 100 °C attribute to the removal of water. The fresh catalyst showed no weight loss above 100 °C (Fig. 10(A)), indicating that it had good thermal stability. However, after the reaction, we can observe from the DTG curve (Fig. 10(B)) that the catalyst had two clear weight losses at 341.7 °C and 605.1 °C, corresponding to different degrees of combustion of acetylene polymer/green oil in air. According to the literature,^41–43^ the weight loss at lower temperatures in the range 200–470 °C is attributed to soft hydrocarbon adsorbed in the catalyst pores or on the surface, which can be removed by treatment at high temperature or solvent extraction, and the weight loss above 470 °C is due to a hard graphite-like hydrocarbon, which can only be removed by oxygen treatment at high temperature. According to the TG curve that the weight losses of the catalyst in these two temperature ranges were 21.8 wt% and 54.7 wt%, respectively; therefore, most of the weight loss occurs above 470 °C, indicating that after repeated regeneration, the deposits on the catalyst surface are mostly graphite-like hydrocarbons that are difficult to remove. Additionally, Table 2 showed the structural parameters of the catalyst before and after reaction 126 h. The specific surface area and pore volume of the catalyst after the reaction 126 h are almost zero, indicating that the catalyst is completely covered by green oil after the reaction 126 h, which is consistent with TG results.

**Table 2:** BET parameter for 1%Ni/MCM-41 before and after reaction 126 h

Catalyst	Surface area (m^2^ g^−1^)	Pore volume (cm^3^ g^−1^)	Pore size (nm)
1%Ni/MCM-41 fresh	793	0.6008	3.03
1%Ni/MCM-41 used	0.0738	0.000551	—

## 4. Conclusions

In summary, Ni/MCM-41 exhibits an excellent catalytic performance in the hydrogenation of pure acetylene to ethylene based on the calcium carbide acetylene route. Under the optimal conditions of 250 °C, 8000 mL g^−1^ h^−1^, and v(H_2_)/v(C_2_H_2_) = 3, acetylene conversion reaches 100%, ethylene selectivity reaches 47%, ethane selectivity is below 10%, and green oil selectivity is around 40%. We found that in this reaction system, in addition to ethylene, green oil is produced in very high yield, and this is the main reason for the low ethylene selectivity. Green oil deposition on the catalyst surface leads to catalyst deactivation. These results provide a strategy for developing Ni-based catalysts for hydrogenation of pure acetylene to ethylene.

## Conflicts of interest

There are no conflicts to declare.

## Supplementary Material
